# Contact Malaria

**Published:** 1916-03

**Authors:** 


**Contact Malaria.** Rist and Rolland, Presse Med., Oct. 1915, report a case in an Alpine Chassur of Savoy who had never been out of the country. He had been frequently stationed near African troops who furnished numerous cases. Without questioning the origin of the plasmodium, this case does not warrant the term ''autochthonous'' which the author employs in the title nor ''contact'' which he employs in the text, unless conveyance by anopheles or in some other analogous manner is eliminated.

